# A clinical assessment of portable point-of-care testing for quick cortisol assay during adrenal vein sampling

**DOI:** 10.1038/s41598-023-49808-5

**Published:** 2023-12-16

**Authors:** Ko Aiga, Mitsuhiro Kometani, Shigehiro Karashima, Seigo Konishi, Takuya Higashitani, Daisuke Aono, Xurong Mai, Mikiya Usukura, Takahiro Asano, Ayako Wakayama, Yuko Noda, Wataru Koda, Tetsuya Minami, Satoshi Kobayashi, Toshinori Murayama, Takashi Yoneda

**Affiliations:** 1 Department of Health Promotion and Medicine of Future, Kanazawa University Graduate School of Medicine, Kanazawa, Ishikawa 920-8641 Japan; 2 Department of Internal Medicine, Houju Memorial Hospital, Nomi, Ishikawa 923-1226 Japan; 3 Department of Radiology, Kanazawa University Graduate School of Medical Sciences, Kanazawa, Ishikawa 920-8641 Japan; 4 Innovative Clinical Research Center, Kanazawa University, Kanazawa, Ishikawa 920-8641 Japan

**Keywords:** Nanobiotechnology, Endocrinology

## Abstract

This study assessed the clinical performance of point-of-care testing (POCT) for quick cortisol assay (QCA) during adrenal vein sampling (AVS) using a newly invented portable quantitative assay instrument. An observational study was conducted prospectively at two centres in Japan. Forty-eight patients with primary aldosteronism considered for adrenalectomy were enrolled in this study and underwent AVS. Three basal adrenal vein samples from each adrenal vein and two from the inferior vena cava were collected sequentially. The cortisol concentration of adrenal vein samples was measured by routine method and QCA. A total of 338 adrenal vein samples were analysed from 250 sites to determine AVS success or failure. The distribution of turnaround time of the QCA for AVS success or failure followed a normal distribution with an average of 20.5 min. A positive correlation between the routine method and QCA was observed regarding cortisol concentration or selectivity index. No significant difference between the two methods was observed regarding the success rate of AVS. Using the routine method as a reference, the sensitivity and specificity of AVS success or failure were 99.1% (210/212) and 81.6% (31/38), respectively. Easy, quick, portable, and precise POCT-QCA demonstrated its compatibility with routine methods regarding clinical performance.

## Introduction

Primary aldosteronism (PA) is the most frequent form of secondary hypertension caused by adrenal tumours with the autonomous secretion of aldosterone^1^. The prevalence of cardiovascular complications is higher in patients with PA than in those with essential hypertension, raising the importance of early detection and appropriate treatment^2,3^. Based on the localization of the lesion, PA is classified into two subtypes, unilateral PA and bilateral PA. The treatment for PA differs depending on the subtypes. Life-long medication of mineralocorticoid receptor antagonists (MRAs) is an optimal treatment for bilateral PA^4^. However, adrenalectomy is the optimal treatment for unilateral PA, which has a potential curative effect^5^. Thus, identification of the PA subtypes is crucial.

While adrenal vein sampling (AVS) has been the gold standard method for subtyping PA, it is cumbersome and challenging. AVS requires a well-equipped facility and radiologists with high expertise or experience^6,7^. Moreover, radiation exposure and complications are risks associated with the procedure^8,9^. The provisional success of AVS is confirmed by imaging the catheter using computed tomography (CT) or angiography, which increases the success rate of AVS^10–12^. In contrast, the technical success of AVS is confirmed by the selectivity index (SI), the ratio of cortisol concentration in the blood of adrenal veins and inferior vena cava (IVC) or peripheral blood^9^. Generally, measuring the cortisol concentration of samples from AVS takes time, and the result comes out after patients’ discharge, which is time-consuming and reduces patients’ quality of life.

In the last few decades, some studies have revealed the benefits of using intraprocedural cortisol measurement (ICM) during AVS, enabling the biochemical confirmation of catheter cannulation in the adrenal veins^13,14^. Based on the accumulation of evidence, the clinical guideline for PA published by the Japan Endocrine Society in 2021 recommends incorporating intraprocedural cortisol measurement during AVS^15^.

Recently, we invented a point-of-care-testing (POCT) for quick cortisol assay (QCA) (POCT-QCA)^16^. The novel POCT-QCA allows us to rapidly measure the cortisol concentrations of the samples drawn by AVS semi-quantitatively or quantitatively, making it possible to confirm the technical success of AVS during the procedure directly. This has led to a significant increase in the success rate of AVS performed by inexperienced radiologists and radiologists who specialize in AVS^16–20^. This multi-centre prospective observational study aimed to investigate the clinical performance of QCA using a new portable quantitative assay instrument by comparing it with the routine AVS measurement.

## Results

### Patients’ characteristics and data collection

Forty-eight patients were enrolled in this study. Table 1 shows the clinical characteristics of the 48 patients before conducting AVS. Samples from 43 patients were diluted and the remaining samples from five patients were measured without dilution. Samples were regarded as unable to determine AVS success or failure when the samples in which dilution was not performed outranged the dynamic range of the QCA. Of the five patients without dilution, two were excluded from the analysis for being unable to determine the AVS success or failure by the QCA. Of the 43 patients with dilution, one was excluded from the analysis due to an insufficient amount of sample drawn by AVS. A total of 353 samples were measured by either the QCA or routine method. The number of IVC and adrenal vein samples that required dilution in the QCA measurement was 83 and 246, respectively. Of the samples measured by the QCA and routine method, the value of cortisol concentration was not determined for 14 samples (13: out of dynamic range of the QCA, 1: complete failure) (Supplementary Table S2). A total of 338 samples from 250 sites were evaluated to determine the AVS success or failure in both the routine and QCA measurements (Supplementary Table S3).

**Table 1:** Clinical characteristics of the Patients before AVS.

	Clinical characteristics (before AVS)
Sex (male/female)	26/22
Age (years)	51.8 ± 9.9
Systolic blood pressure (mmHg)	136.3 ± 13.9
Diastolic blood pressure(mmHg)	82.4 ± 10.8
Serum potassium (mEq/L)	3.9 ± 0.3
PRA (pg/mL/hr)	0.4 ± 0.3
PAC (pg/mL)	189.6 ± 131.5

*AVS* Adrenal vein sampling, *PRA* Plasma renin activity, *PAC* Plasma aldosterone concentration.

### Sensitivity and specificity of the success/failure of insertion into adrenal veins during the AVS in QCA

The definitions of the sensitivity and specificity of insertion into adrenal veins during the AVS in QCA are defined in Supplementary Table S3. The sensitivity and specificity were 99.1% (210/212) and 81.6% (31/38), respectively (Table 2). The chi-squared test did not show AVS success or failure dependency on cortisol measurement kits, indicating no significant differences in AVS success rate between the QCA and routine method.

**Table 2:** Sensitivity and specificity of QCA.

	Categorical/statistical value	AVS success or failure (Routine method)		
		Success	Failure	Total
Determination of AVS success or failure in both kits (QCA and Routine method)		212	38	–
AVS success/failure (QCA)	Success	210	7	217
	Failure	2	31	33
	Total	212	38	250
QCA sensitivity (QCA success/Routine method success)	Point estimation (%)	99.1	–	–
	Confidence interval (95%)	(96.6, 99.9)	–	–
QCA specificity (QCA failure/Routine method failure)	Point estimation (%)	–	81.6	–
	Confidence interval (95%)	–	(65.7,92.3)	–

*AVS* Adrenal vein sampling, *QCA* Quick cortisol assay.

### Correlations between routine method and QCA

**Figure 1:**
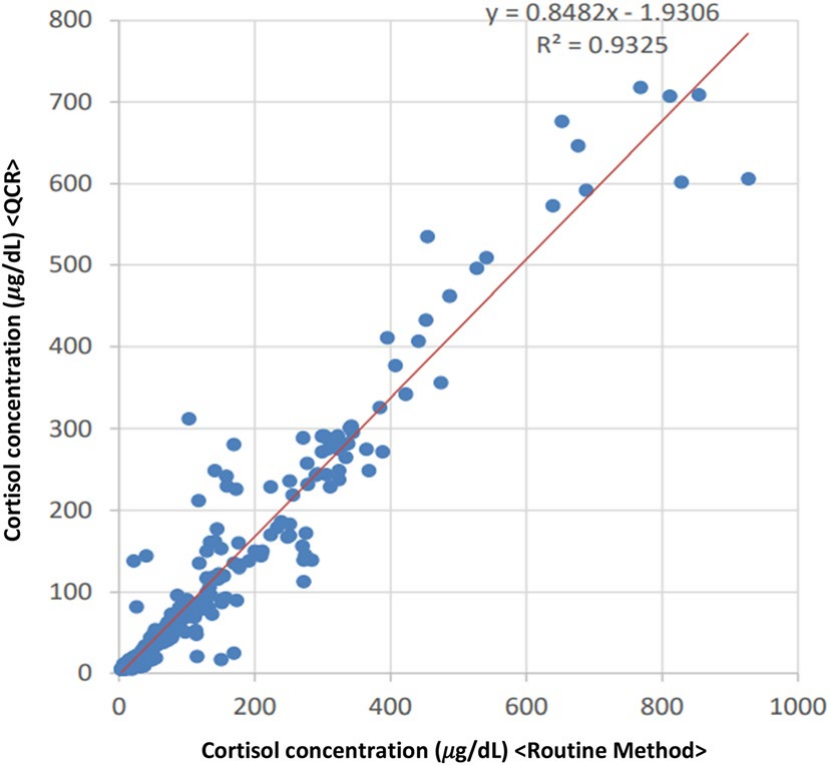
The scatter plot of cortisol concentration (QCA versus routine method). Of the adopted sites of cortisol concentration measurement, samples that were lower than the upper limit of the QCA and routine method were plotted (n = 330). The X-axis represents the value of cortisol concentration measured by the routine method. The Y-axis represents the value of cortisol concentration measured by the QCA. The red line represents the regression line.

**Figure 2:**
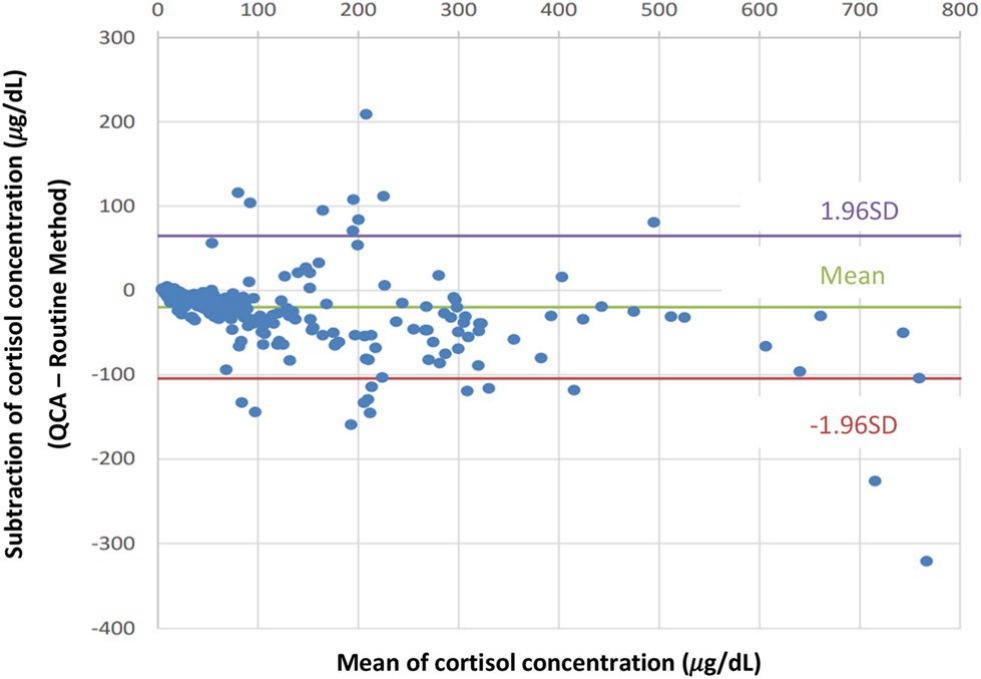
The Bland–Altman plot of the cortisol concentration (QCA versus routine method). Of the adopted sites of cortisol concentration measurement, samples that were lower than the upper limit of the QCA and routine method were used to plot the Bland–Altman plot (n = 330). The purple/red line represents a 95% confidence interval. The green line represents the mean value.

Cortisol concentration was analysed statistically by two types of correlation analysis. Figure 1 shows the correlation between cortisol concentration using the routine method and QCA. The Pearson correlation coefficient was 0.966, and the Spearman correlation coefficient was 0.962. Both results showed a positive correlation in cortisol concentration. A tendency for the cortisol concentration to be higher in the routine method than in QCA was observed. Figure 2 shows the Bland–Altman plot of cortisol concentration for the routine method and QCA. Pearson and Spearman correlation coefficients showed a negative correlation between the subtraction value of cortisol concentration and the mean value of cortisol concentration (− 0.448 and − 0.632, respectively). Plots outside the 95% confidence interval (CI) limits of agreement were observed, and a discrepancy between the routine method and QCA in cortisol concentration was confirmed.

**Figure 3:**
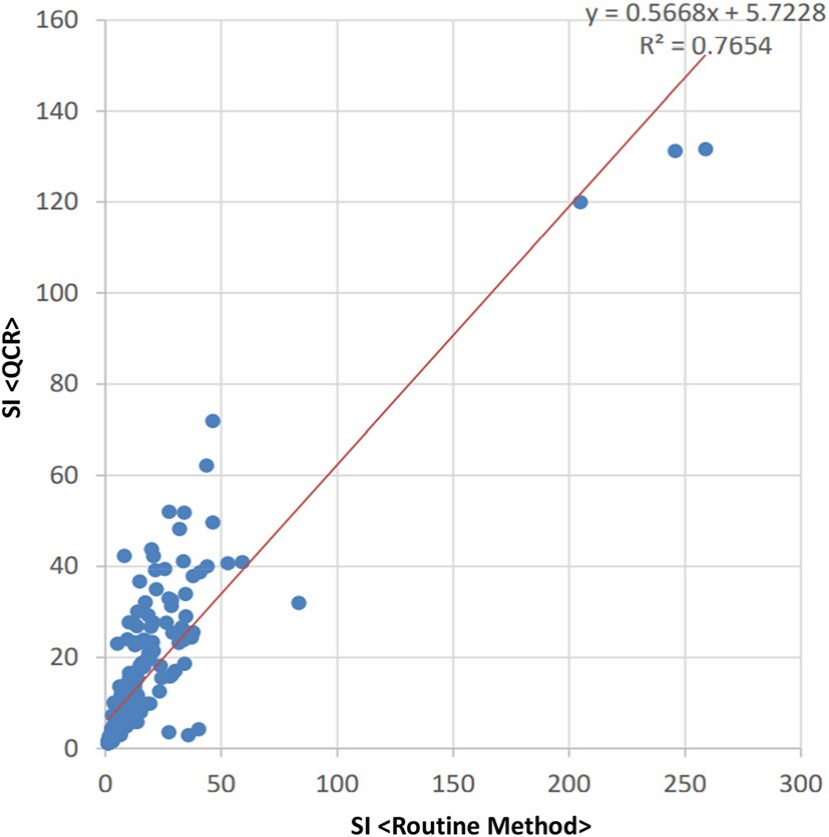
The scatter plot of SI (QCA versus routine method). Of the adopted sites of cortisol concentration measurement, samples that were lower than the upper limit of the QCA and routine method were used to calculate the SI and plot the scatter plot of the SI (n = 242). The X-axis represents the value of SI computed by measurements from the routine method. The Y-axis represents the value of SI computed by measurements from the QCA. The red line represents the regression line.

**Figure 4:**
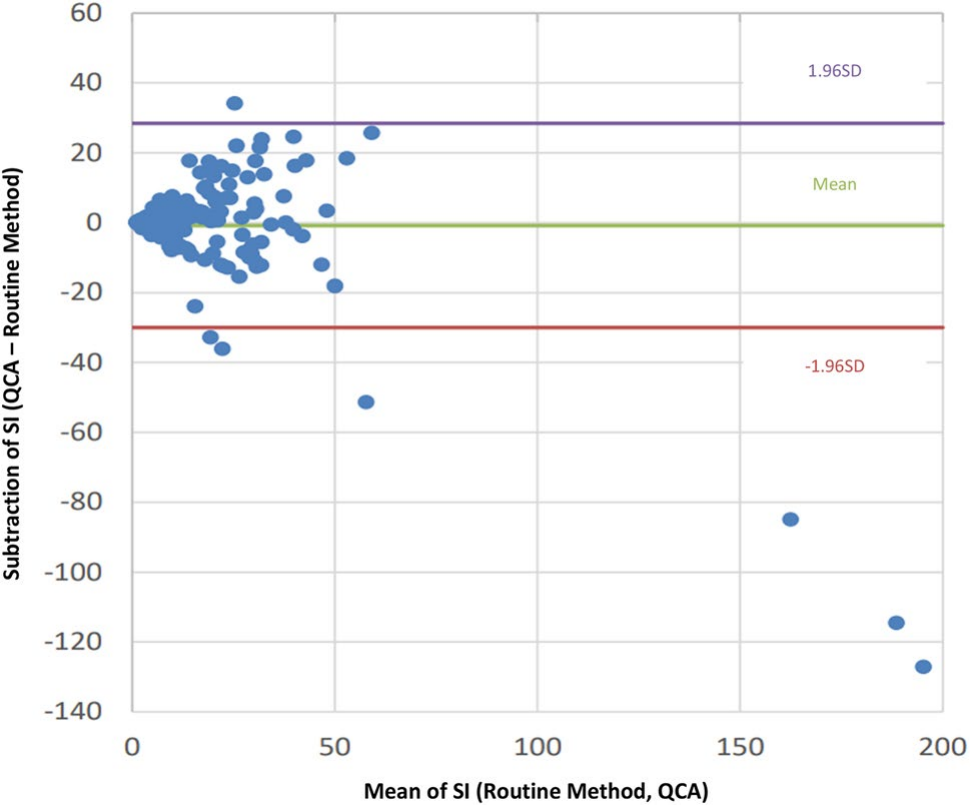
The Bland–Altman plot of the SI (QCA versus routine method). Of the adopted sites of cortisol concentration measurement, samples that were lower than the upper limit of the QCA and routine method were used to calculate the SI and were used to plot the Bland–Altman plot (n = 242). The purple/red line represents a 95% confidence interval. The Green line represents the mean value.

The correlation between the SI of the routine method and QCA was investigated by two types of correlation analysis (Pearson and Spearman). Figure 3 shows the correlation between the SI of the routine method and QCA. The Pearson correlation coefficient was 0.875, and the Spearman correlation coefficient was 0.898. Both results showed a positive correlation in SI. A tendency for the SI to be higher in the routine method than in QCA was observed. Figure 4 shows the Bland–Altman plot of SI for the routine method and QCA. A negative correlation was observed between the subtraction value of the SI and the mean value of the SI in the Pearson correlation coefficient (− 0.679). However, no correlation was observed using the Spearman correlation coefficient (0.118). Plots outside the 95% CI limits of agreement were observed, and a discrepancy between the routine method and QCA regarding SI was confirmed.

### Turnaround time of AVS

**Figure 5:**
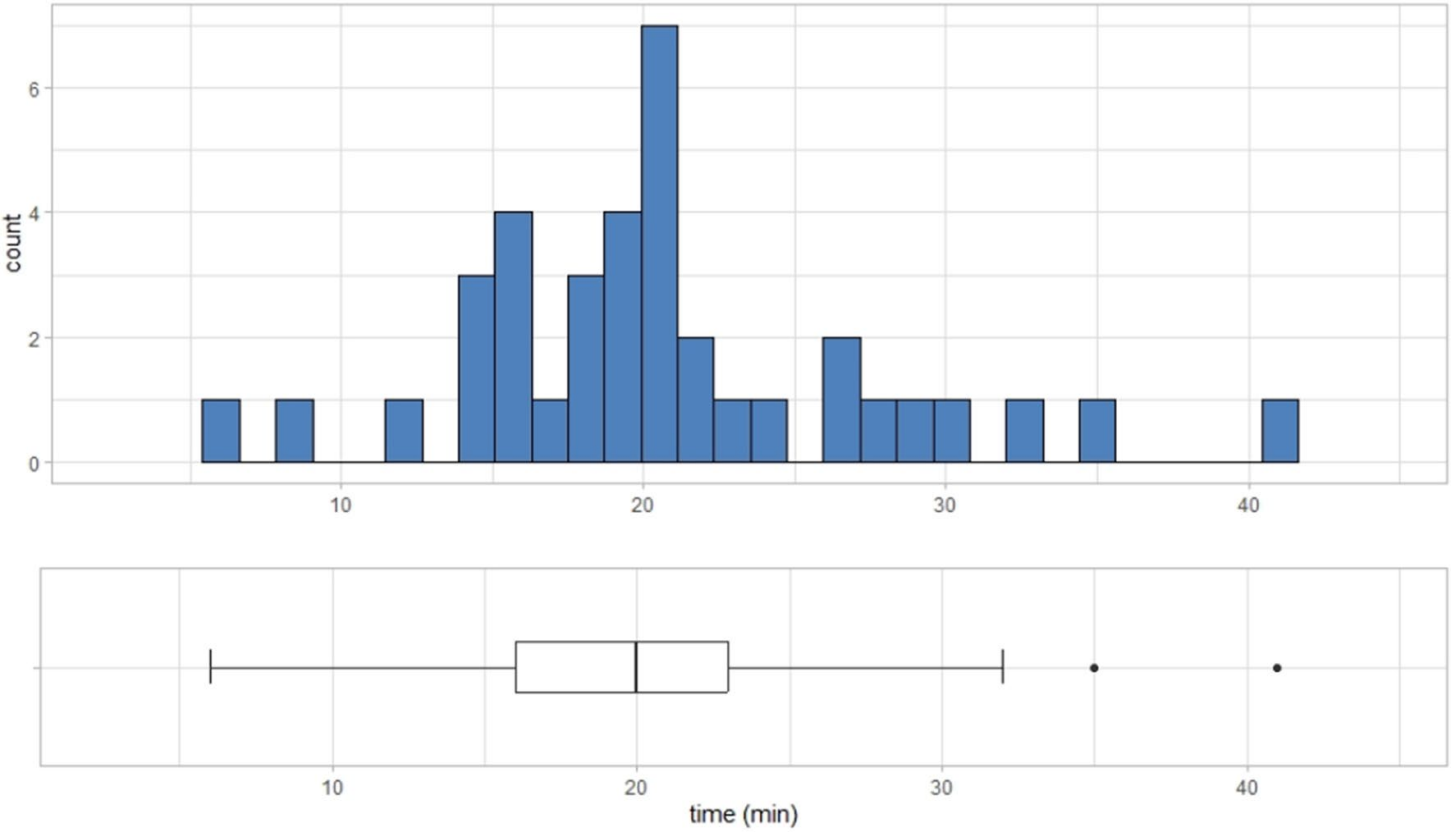
The turnaround time of the AVS. The turnaround time was defined as the time for the first right adrenal vein samples to determine AVS success or failure after taking IVC samples. The histogram above shows the distribution of the turnaround time. A box plot of the turnaround time was drawn below the histogram.

Of the 45 cases included in the analysis, sampling was performed from the IVC, right adrenal vein, and left adrenal vein, in that order, except in six cases. These six cases were excluded when analysing the turnaround time. The average turnaround time was 20.5 min (95% CI 18. 2 $\le \overline{x}\le$ 22.9 min). Figure 5 shows the boxplot and corresponding distribution of the turnaround time. The p-value of the Shapiro–Wilk normality test for the distribution of the turnaround time was 0.097 (> 0.05), suggesting a normal distribution. The number of dilutions required to determine the turnaround time was 1 or 0, and the average was 0.83.

### Adverse events

An adverse event was confirmed in only one case; the rate was 2.1% (1/48). It was observed during AVS due to vagal response. However, the patient recovered after atropine sulphate hydrate was administered, and recovered.

## Discussion

This study investigated the clinical performance of QCA during AVS. The result of AVS was rapidly available with small fluctuations in measuring time. High sensitivity and specificity were confirmed in the QCA. Additionally, the routine method observed a positive correlation between cortisol concentration and SI. These results demonstrated that QCA is clinically comparable to routine cortisol measurement.

Recently, bypassing AVS before surgical treatment of unilateral PA has been controversial. According to the Japan Endocrine Society clinical practice guideline, AVS is the gold standard subtyping technique for PA^4,15^. However, some literature argues that patients with typical PA findings or PA at an early age may be considered for adrenalectomy without AVS^15,21–24^. Circumstances behind the argument could be lack of standardization, costs, complication risks, and low success rate of AVS^6,8,21,25^. Currently, several alternative tests for subtyping PA are proposed in the literature. Karashima et al.^26^ recently developed an artificial intelligence (AI) model based on ensemble learning to predict the subtypes of PA. The model made it possible to predict the subtype of PA from the basic clinical information of patients. However, AVS needs to be conducted to determine the dominant gland of the unilateral PA. Recently, non-invasive imaging tests have been developed in PA localization. Wu et al. reported that imaging results using metomidate were as accurate as those obtained by AVS^27^. However, it should be noted that metomidate binds to CYP11B1, involved in cortisol production, and CYP11B2. Furthermore, the very short half-life of Carbon-11 radionuclide of 20 min is a major issue in actual use. Other imaging modalities targeting the chemokine receptor 4 (CXCR4), an inflammatory cytokine^28^, and compounds that specifically bind to CYP11B2 are also in development^29^. However, sufficient clinical evidence is still lacking. Among the alternative tests, AVS is the most reliable localizing test for PA^21,30^. Therefore, it is important to establish and improve AVS.

ICM has been regarded as one of the solutions to the difficulty experienced in AVS. Several studies have proved that resampling until successful insertion into the adrenal vein significantly improved the success rate of cannulation in the right adrenal vein with a low rate of complications during the procedure^31–34^. In addition, receiving feedback in real-time allowed us to self-train, reducing the rate of resampling experienced by the radiologist when using ICM^31,32^. One study also reported that ICM reduced the amount of radiation exposure^35^. However, ICM has been cumbersome and stressful for radiologists and patients. ICM often requires that patients be moved to the recovery room from the radiology suite while samples are transferred and analysed in the laboratory room^34,36^.

Furthermore, the turnaround time of cortisol measurement is long (20–120 min)^18,32–34,36,37^. These procedures make patients uncomfortable, and moving between the same facility’s radiology suite and laboratory room increases the financial burden. Our new POCT-QCA enables us to perform ICM rapidly in a simple process with low costs and high success rates^16,18,19^. As the ICM reported previously, studies have revealed that our QCA improves radiation exposure during AVS and the success rate of AVS^20^.

This study investigated the clinical performance of QCA. No significant difference in the AVS success rate was observed between the routine method and QCA. Additionally, specificity and sensitivity were 210/212 (99.1%) and 31/38 (81.6%), respectively, and a high positive correlation was observed between routine method and QCA in cortisol concentration and SI (Pearson correlation coefficient or Spearman correlation coefficient in both cortisol concentration and SI was > 0.875). These results validated the consistency of the AVS results between the routine method and QCA. However, the Bland–Altman plot showed disagreement between the routine method and QCA in cortisol concentration and SI, indicating a discrepancy in the absolute cortisol concentration and SI between the routine method and QCA. Although the absolute values measured by the QCA did not show similarity to those of the routine method, the performance of the QCA during AVS is clinically comparable to that of the routine method.

The results also demonstrated additional advantages in incorporating our QCA clinically. The average turnaround time was 20.5 min, relatively quick compared to the previously reported ICM (20–120 min)^18,32–34,36,37^. The average dilutions required to determine AVS success or failure was 0.83. Zero or one dilution was sufficient to turn around the AVS results. This result reflected the stability of the turnaround time and followed a normal distribution. Access to the rapid turnaround of AVS could reduce patients’ discomfort. At the same time, the simple process with fewer dilutions could help clinicians avoid making mistakes. Our QCA may be beneficial to both healthcare providers and patients.

The clinical performance of QCA evaluated in this study may be assessed not only by quantitative assay methods but also by semi-quantitative assay methods. When using the SI of 2 or higher criterion in AVS without adrenocorticotropic hormone (ACTH) load, the semi-quantitative assay, as reported previously, would be effective^16^. However, in the semi-quantitative assay of QCA, the decision line almost disappears at concentrations of 552–828 nmol/L or higher, as reported previously^16^. Therefore, it is difficult to semi-quantitatively evaluate the result in patients with high cortisol levels at IVC. Furthermore, ACTH-stimulated AVS is recently being performed^15^, and as observed, in ACTH-stimulated AVS, the cortisol concentration in IVC is higher, and the SI criterion used is also higher than five. Under these circumstances, it is difficult to reliably determine the success or failure of insertion into the adrenal vein using only the semiquantitative assay. A quantitative assay, in addition to dilution, is a reliable method for these cases.

In summary, we assessed the clinical performance of POCT-QCA. Using the QCA, the result of AVS was available in a short time. Furthermore, high specificity and sensitivity and a positive correlation between the routine method and QCA were observed regarding cortisol concentration and SI. This portable and user-friendly QCA can be useful clinically.

## Methods

### Clinical studies

This multicentre prospective observational study was performed at Kanazawa University Hospital and Houju Memorial Hospital from June 2019 to February 2021. This study was performed in accordance with the guidelines for clinical research published by the Japanese Ministry of Health, Labour and Welfare.

All participating patients provided informed consent. The ethics committee of both Kanazawa University School of Medicine and Houju Memorial Hospital approved this study. This study was conducted in collaboration with Kanazawa University and Trust Medical Co., Ltd. (Kasai, Hyogo, Japan), and registered in University Hospital Medical Information Network Clinical Trials Registry (ID: UMIN000037157). All the procedures of this study were performed in accordance with the Helsinki Declaration.

### Patients

This study involved 48 patients diagnosed with PA and older than 20 years, in whom adrenalectomy was considered for treatment. All the diagnoses adhered to the guidelines of the Japan Endocrine Society or Japanese Society of Hypertension^15,38^.

### AVS procedure

Before AVS, anti-hypertensive drugs were substituted with calcium channel blockers or alpha 1 blockers to exclude the effects of medication on plasma aldosterone concentration (PAC) or serum cortisol concentration^39^. Samples were collected sequentially from three points; the IVC, right adrenal vein, and left adrenal vein. AVS was performed using the following process:

1. A catheter was inserted in the IVC from the right or left femoral vein cava.
2. AVS was performed at the IVC, right adrenal vein, and left adrenal vein, in that order. After the cannulation of each adrenal vein, samples were collected three times before switching to the other adrenal vein.

If it was difficult to insert the right adrenal vein during the procedure, the left adrenal vein sample was drawn in advance and then, a re-attempted drawing of the right adrenal vein sample was done. During the procedure, catheter insertion was provisionally confirmed by imaging (including venography, X-ray fluoroscopy, and CT).

### Cortisol measurement using QCA

1. Mechanism of QCA and immunochromato reader

**Figure 6:**
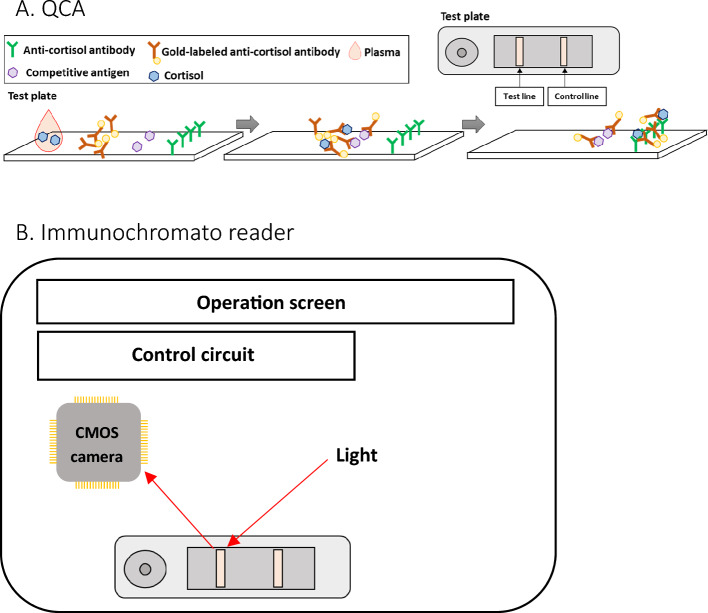
The outlines of the QCA and immunochromato reader (**A, B**). (**A**) The test plate and the internal structure of the QCA. Competitive antigens and anti-cortisol antibodies are arrayed at the immunochromatographic paper’s test and control lines, respectively. Gold nanoparticle labelled anti-cortisol antibodies are fixed in the drip site. (**B**) Immunochromato reader. The CMOS camera measures the brightness of the reflection from the gold nanoparticles on the test line of the test plate using the light source. The control circuit regulates the output of the CMOS camera.

QCA (Quick cortisol kit, Q-CTZ-1000, Trust Medical Co., Ltd., Kasai, Hyogo, Japan), an immunochromatographic assay, was used to measure cortisol concentration during AVS (width: 70 mm, depth: 18 mm, height: 5 mm). The mechanism of QCA is shown in Fig. 6. The components of the QCA included gold-labelled anti-cortisol monoclonal antibodies, competitive antigens, and anti-cortisol monoclonal antibodies. The gold-labelled antibodies, competitive antigens, and antibodies were attached to the conjugate pad, test line, and control line. Cortisol molecules in the plasma bonded with the gold-labelled antibodies to become antigen–antibody complex after adding plasma to the test plate of the QCA. The competitive antigens caught the remaining gold-labelled antibodies in the control line. The antigen–antibody complexes were trapped in the control line by the anti-cortisol antibodies. Immunochromato reader (TOR 210, Trust Medical Co., Ltd. Kasai, Hyogo, Japan) is a portable device (Supplementary Fig. S1; width: 260 mm, depth: 168 mm, height: 86 mm, weight: 750 g) that measures the brightness of the lights reflected by the nano-gold particles in the test line of the test plate using a CMOS camera, with user-friendly interface. The calibration curve of brightness and cortisol concentration computed cortisol concentration.

(2) QCA measurement steps

Using QCA, cortisol concentration was measured quantitatively.

The procedure of QCA was performed as follows:

1. A 1.5 mL blood sample drawn by AVS was centrifuged at 10,000 rpm for 1 min.
2. QCA was set on the immunochromato reader (Immunochromato reader, TOR210, Trust Medical Co., Ltd. Kasai, Hyogo, Japan).
3. 100 μL of plasma was added to the QCA.
4. 10 min after plasma was added into the QCA, the brightness of chemiluminescence in the QCA was measured using the immunochromato reader.

A sample with a high cortisol concentration that outranged the upper limit of the QCA dynamic range (5–20 μg/dL) was diluted using rabbit serum. A sample from the IVC underwent a twofold dilution. If the cortisol concentration in the IVC sample was below the QCA dynamic range, the cortisol concentration value for SI calculation was set at 5 μg/dL.

Samples from adrenal veins, whose cortisol concentration was higher than the QCA dynamic range, were diluted and remeasured. Samples from adrenal veins underwent a tenfold dilution initially. If an additional dilution was needed, the sample underwent a 2, 3, 5, 10, 20, or 40-fold dilution, depending on the result of the remeasurement.

Complete failure of QCA measurement was defined as a failure in measurement due to the technical problems of QCA after three attempts.

Turnaround time, defined as the time to assess the technical success of AVS after taking the IVC samples, was measured. Only the turnaround time for the initial right adrenal vein sample was recorded and analysed.

### Routine cortisol measurement

Routine cortisol measurement was done using the conventional cortisol assay (Roche Elecsys® cortisol II assay, Roche Diagnosis). This widely used assay uses electrochemiluminescence immunoassay (ECLIA), which uses monoclonal assay to measure serum cortisol levels. This method can be traced to a gas chromatography-mass spectrometry/mass spectrometry (GC–MS/MS) reference^40,41^. Serum cortisol levels ranging between 7.07 and 19.6 μg/dL can be reliably measured using this assay^42^.

### AVS success criteria

AVS success or failure and the diagnosis of PA subtypes were assessed based on the cortisol concentration gained from routine measurement. We defined AVS success as SI > 2 for the routine cortisol measurement (SI: ratio of cortisol concentration from the adrenal vein sample and cortisol concentration from the IVC sample). This criterion is consistent with the Endocrine Society guidelines^4^.

### Statistical analysis

The sensitivity and specificity of success/failure of insertion into adrenal veins during the AVS in QCA were calculated based on the assessment of routine cortisol measurement. The definitions of specificity and sensitivity are shown in Supplementary Table S3. The SI or cortisol concentration correlation between the routine method and QCA was evaluated using the equation: y = ax + b. Additionally, the Bland–Altman plot method was used to evaluate the comparison of either SI or PAC between routine cortisol measurement and QCA. These statistical analyses and figures (Figs. 1, 2, 3, and 4) were made by EPS Corporation (Shinjuku, Tokyo). In addition, we analysed the turnaround time of the AVS and the error analysis between the routine method and QCA using R software (version 4.1.3).

### Supplementary Information

Supplementary Information.

## Data Availability

The datasets generated and analysed during the current study are not publicly available due to ethical restrictions but are available from the corresponding author on reasonable request.
