# Supplementary material for: A clinical assessment of portable point-of-care testing for quick cortisol assay during adrenal vein sampling
Source: Sci Rep. 2023 Dec 16;13:22429. doi: 10.1038/s41598-023-49808-5 (PMC10725449; doi:10.1038/s41598-023-49808-5)
Supplement: Supplementary file 1 — Supplementary Information. [file 41598_2023_49808_MOESM1_ESM.docx]

**Supplemental Table**

**Supplemental Table S1. Cortisol concentration at non-adapted sites**

| Cortisol concentration measurement (non-adopted sites) | | | | | |
| --- | --- | --- | --- | --- | --- |
|  | | QCA | | |  |
|  |  | No dilution  N = 3 | Dilution  N = 42 | Total  N = 45 | Routine Method  N = 45 |
| Sites | Reasons |  |  |  |  |
| IVC | Upper limit < | 1 | - | 1 | - |
|  | Uncertainty of measured value | 0 | 0 | 0 | 0 |
|  | Total | 1 | 0 | 1 | - |
| SI | Upper limit < | 12 | - | 12 | - |
|  | Uncertainty of measured value | 0 | 1 | 1 | 1 |
|  | Total | 12 | 1 | 13 | 1 |

QCA, quick cortisol assay; IVC, inferior vena cava; SI, selectivity index.

**Supplemental Table S2. Cortisol concentration measurement (adopted sites)**

| Cortisol concentration measurement (adopted sites) | | | | | |
| --- | --- | --- | --- | --- | --- |
|  | QCA | | |  |  |
|  | No dilution  N = 3 | Dilution  N = 42 | Total  N = 45 | Routine Method  N = 45 | Both  (QCA & Routine method) |
| Sites |  |  |  |  |  |
| IVC | 5 | 83 | 88 | 89 | 88 |
| SI computed sites | 6 | 245 | 251 | 263 | 250 |
| Total | 11 | 328 | 339 | 352 | 338 |

QCA, quick cortisol assay; IVC, inferior vena cava; SI, selectivity index.

**Supplemental Table S3. Definition of sensitivity and specificity**

|  | AVS Success (QCA) | AVS Failure (QCA) | Total |
| --- | --- | --- | --- |
| AVS Success (Routine) | A | C | A + C |
| AVS Failure (Routine) | B | D | B + D |
| Total | A + B | C + D | A + B + C + D |

AVS, adrenal vein sampling; QCA, quick cortisol assay.

Sensitivity = $\frac{A}{A+C}$ , Specificity = $\frac{D}{B+D}$

**Supplemental figure S1. Immunochromato reader (TOR 210, Trust Medical Co.,Ltd. , Kasai, Hyogo, Japan)**


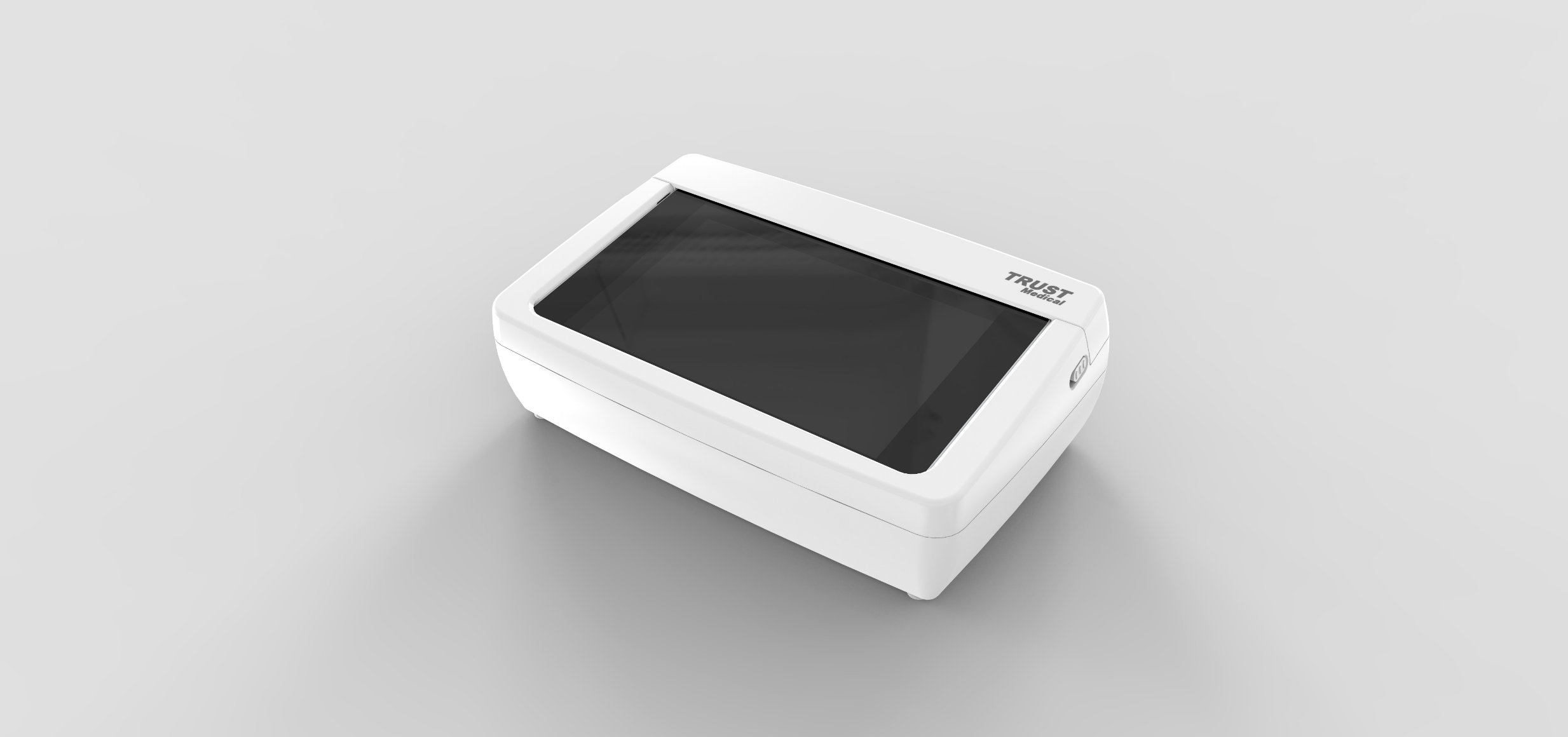


Width: 260 mm, Depth: 168 mm, Height: 86 mm, Weight: 750 g.
